# Adjustment to the learning environment among university students who are deaf or hard of hearing

**DOI:** 10.4102/sajcd.v72i1.1114

**Published:** 2025-09-30

**Authors:** Kayla Percival, Mahanoor Ahmed, Nasim B. Khan

**Affiliations:** 1 Discipline of Audiology, School of Health Sciences, University of KwaZulu-Natal, Westville Campus, Durban, South Africa

## Abstract

**Background:**

Ensuring students who are deaf/Deaf (d/Deaf) or hard of hearing (d/DHH) have access to higher education goes beyond legal compliance but reflects an institution’s commitment to creating educational environments where all learners can fully participate and develop.

**Objectives:**

This study explored instructional, psychosocial, and environmental factors influencing adjustment to the learning environment among d/DHH students at the University of KwaZulu-Natal (UKZN).

**Method:**

A qualitative design was employed, with semi-structured interviews conducted with 10 purposively sampled d/DHH students across four UKZN campuses (Westville, Pietermaritzburg, Howard College, and Edgewood). UKZN, one of South Africa’s largest and most diverse universities, is recognised internationally for its focus on inclusive education and transformation.

**Results:**

Thematic analysis revealed eleven themes, including disclosure of disability, interpreter accessibility, classroom acoustics, and attitudinal barriers. Disclosure was vital for accessing support services, while interpreters were key enablers of participation, though mainly available during lectures. Poor classroom acoustics, lighting, noise, and rapid speech negatively affected interpretation. Attitudinal barriers, limited awareness of Deaf culture among peers, and inflexible teaching practices contributed to alienation and isolation. Nonetheless, supportive lecturers and peers facilitated better adjustment, highlighting the role of inclusive behaviours in enhancing learning experiences.

**Conclusion:**

While positive examples of accommodation were noted, significant challenges remain. The findings suggest the need for comprehensive strategies, including awareness, staff training, improved infrastructure, accessible technology, and strengthened disability support units, to foster inclusive environments that promote full participation of d/DHH students in higher education.

**Contribution:**

Addressing instructional, psychosocial and environmental barriers is essential for ensuring equitable access to higher education and academic success for d/DHH students. By examining the lived experiences of these students, this study provides valuable insights to inform more effective and inclusive institutional strategies.

**Keywords:**

deaf/Deaf or hard of hearing; tertiary education; accessibility; inclusion; support services; communication barriers; Deaf culture; educational accommodations.

## Introduction

The Constitution of the Republic of South Africa (1996) legally mandates the right to education for all individuals, regardless of race, class, gender, culture, religion, language or disability (Mutanga, 2018). In South Africa, enabling and progressive legislation for persons with disabilities facilitated and culminated in the increased enrolment of university students who are d/Deaf or hard of hearing (d/DHH)^1^ (Bell & Swart, 2018). However, d/DHH students remain underrepresented, comprising less than 1% of the student population at tertiary institutions (Bell & Swart, 2018).

The conceptualisation of disability in higher education has evolved through the influence of the global Disability Rights Movement, which has shaped both societal expectations and legislative mandates aimed at promoting accessibility (McDaniel, 2018). In South Africa, the framework for disability rights is grounded in human rights values, as outlined in the Constitution of the Republic of South Africa (*Act No. 108 of 1996*). This legal document enshrines the rights of all individuals in the country and upholds democratic values such as human dignity, equality and freedom (África, 2020). The *White Paper on an Integrated National Disability Strategy* (1997) reinforces this foundation by advocating for a rights-based approach, the adoption of the social model of disability and the inclusion of persons with disabilities in decision-making processes (Munzhedzi, 2021). Although legal frameworks govern the provision of reasonable accommodations in higher education, true accessibility requires institutional commitment to fostering inclusive and enabling learning environments (Hamraie, 2016).

Two dominant theoretical frameworks continue to shape institutional responses to disability: the medical model, which frames disability as an individual deficit requiring correction, and the social model, which highlights structural barriers and the need for systemic change (Chapman, 2021; Hamraie, 2016; Titchkosky, 2011; Zaks, 2023). The cultural model further conceptualises disability as a valuable aspect of human diversity, particularly relevant within the Deaf and hard of hearing (HoH) community, whose identities are formed through shared language and cultural norms (Cawthon & Garberoglio, 2021; Turkestani & Albash 2022). The educational context plays a central role in shaping and affirming these identities through inclusive curricula, peer relationships and appropriate communication methods (Duncan et al., 2021; Ryan & Junker, 2019).

An integrated biopsychosocial and cultural model is increasingly advocated in higher education settings. This approach acknowledges the interplay between individual health conditions and environmental factors and promotes accessibility through intentional, inclusive design that accommodates diverse needs beyond formal diagnoses (Donald & Frank, 2023; Hamraie, 2016; Petasis, 2019). The socio-ecological model, as proposed by Bronfenbrenner, highlights the importance of examining the support and development of students within the context of their environment through collaboration. Achieving true inclusion requires a concerted effort from all levels, including home and community, to ensure students with disabilities can access the resources and support that they require to thrive in the university environment (Ainscow & Sandill, 2010, cited in Akoto et al., 2022). However, in regions such as sub-Saharan Africa, persistent barriers such as inadequate teaching and learning materials, inaccessible infrastructure and limited teacher training continue to undermine inclusive efforts (Nketsia et al., 2013, as cited in Akoto et al., 2022).

Students in higher education who are d/DHH and who do access tertiary training institutions face significant barriers that hinder their academic success and social integration. Challenges include insufficient psychological support services, communication difficulties, educational and environmental factors (Bell & Swart, 2018; Cawthon et al., 2016). These students struggle with inaccessible curricula, inadequate academic support, stigma and self-esteem issues (Ozcan, 2021). Fear of discrimination often leads to non-disclosure of hearing impairments, further affecting their academic performance (Sekoto & Hlayisi, 2023).

The lack of inclusive policies, faculty training and mentorship programmes exacerbates disparities in educational opportunities (Bell & Swart, 2018; Hameed & Ul-Ain, 2020). Students who are d/DHH face social isolation because of exclusion from extracurricular activities, leaving them disconnected from university life (Parvez et al., 2019). Many students report feeling detached and disengaged from university life, and the academic effort required often leaves little energy for social engagement (Kermit & Holiman, 2018). Some of these students are also unaware of disability support services because of ineffective communication and promotion of available services (Akoto et al., 2022; Cawthon et al., 2016; Kabtyimer, 2020; Yousif et al., 2021).

Inclusive education requires accessibility, reasonable accommodations and targeted support, including assistive devices and faculty collaboration (Jokinen, 2018). Although access to higher education has improved, many of those who do gain access struggle to graduate successfully, often because of a lack of targeted support for their unique communication and learning needs. This highlights the urgent need for comprehensive interventions to promote academic success and meaningful inclusion for d/DHH students (Bell et al., 2016). Addressing these barriers is essential for ensuring equitable access to higher education for students who are d/DHH (Yeager, 2018).

Limited access to higher education restricts career opportunities, reduces earning potential and exacerbates economic inequality (Garberoglio et al., 2021; Zaback et al., 2012). Research on the experiences of d/DHH students in South African universities is scarce, creating gaps in educational, environmental and psychosocial support (Bell & Swart, 2018; Hameed & & Ul-Ain, 2020; Moloi & Motaung, 2014; Oppong et al., 2018). Universities must improve teaching and support structures to enhance students’ academic experiences and future economic independence (Ndlovu & Walton, 2016).

The expertise of audiologists regarding hearing loss and communication can inform tailored accommodations, enhance academic support and contribute to policy development within tertiary training institutions (Cawthon & Garberoglio, 2021). Audiologists play a vital role in assessing and improving the learning environment for students who are d/DHH at the University of KwaZulu-Natal (UKZN). Equitable education improves employment prospects, economic growth and social well-being (Garberoglio et al., 2019; Tsimpida et al., 2018). Addressing these challenges will foster inclusive learning environments, promote cultural pride and support student success (Swart & Pettipher, 2018).

The above rationale led to the development of the following research question: What instructional, psychosocial and environmental factors influence the adjustment of students who are Deaf or HoH attending the University of KwaZulu-Natal?

## Research methods and design

### Study aim and design

This study aimed to explore the instructional, psychosocial and environmental factors influencing adjustment to the learning environment for d/DHH students at the University of KwaZulu-Natal.

The following objectives were developed in order to meet the aim of this study:

- To describe the ***instructional/educational factors*** influencing the adjustment of Deaf or HoH students attending the UKZN.
- To determine the ***psychosocial and cultural factors*** influencing the adjustment of students who are Deaf or HoH attending the UKZN.
- To ascertain the ***environmental/physical factors*** influencing the adjustment of students who are Deaf or HoH attending the UKZN, using a descriptive qualitative research design with elements of the phenomenological approach, the study sought to understand the live experiences of these students. Semi-structured interviews were conducted using an interview schedule that included open-ended questions to gather data. Qualitative research provided a deeper understanding of the factors affecting their educational, environmental and psychosocial adjustments within a South African university context (Alhazmi & Kaufmann, 2022).

### Study setting

The study was conducted at multiple UKZN campuses in KwaZulu-Natal Province, specifically Howard College, Edgewood, Pietermaritzburg (PMB) and Westville campuses.

The sample comprised 10 students diagnosed as d/DHH and registered with the UKZN Disability Unit. Inclusion criteria required participants to be enrolled at the UKZN (full-time or part-time), aged 18–35 years, and documented as students who are d/DHH. The sample included individuals across all genders and academic levels (undergraduate, postgraduate and doctoral). The inclusion of participants aged 18–35 years across all academic levels was intentional to capture a broad spectrum of experiences among students who are d/DHH within the university context. This age range aligns with the demographic composition of many South African tertiary institutions, where students often enter higher education at different life stages because of varied educational, social or economic pathways. Including individuals across academic levels and genders allowed for a richer, more nuanced understanding of how adjustment to university life may differ depending on age, level of study and life responsibilities. This variation also enabled the exploration of intersecting factors such as maturity, academic experience and support needs that influence the lived experiences and coping strategies of d/DHH students in higher education. Participants using hearing aids, cochlear implants or other assistive devices were identified in the recruitment process. Individuals with normal hearing or undocumented hearing loss were excluded.

All demographic information has been captured in Table 1. The sample comprised predominantly female participants (80%, *n* = 8). Most participants (60%, *n* = 6) were enrolled in the School of Education. Half of the participants (50%, *n* = 5) were in their fourth year of study, and the majority (80%, *n* = 8) were pursuing undergraduate degrees. Most participants (80%, *n* = 8) lived in on-campus accommodation, while the remainder resided in private housing (10%, *n* = 1) or at home (10%, *n* = 1). Hearing loss was reported to have occurred at birth (40%, *n* = 4) or between the ages of 4 and 6 years (40%, *n* = 4). The origin of hearing loss was equally distributed between congenital (50%, *n* = 5) and acquired (50%, *n* = 5). The severity of hearing loss was unknown for 60% (*n* = 6), while 20% (*n* = 2) had severe hearing loss, 10% (*n* = 1) had moderate–severe hearing loss and 10% (*n* = 1) had mild hearing loss. Regarding amplification, 80% (*n* = 8) of participants used hearing aids, while 20% (*n* = 2) had cochlear implants. All participants relied on total communication. Parental hearing status showed that 90% (*n* = 9) had hearing parents, with 30% using oral communication and 20% using sign language.

**TABLE 1:** Demographic information of the participants.

Category	Subcategory	*n*	%
Age (years)	19	1	10
	21	1	10
	22	2	20
	23	3	30
	24	1	10
	25	1	10
	27	1	10
Gender	Female	8	80
	Male	2	20
Discipline of study	College of Humanities: School of Social Sciences	1	10
	College of Humanities: School of Education	6	60
	College of Humanities: School of Arts	2	20
	College of Law and Management	1	10
Year of study	1st	2	20
	3rd	2	20
	4th	5	50
	7th	1	10
Degree programme	Undergraduate	8	80
	Masters	1	10
	Postgraduate	1	10
Current degree	Bachelor of Social Science	1	10
	Bachelor of Education	6	60
	Bachelor of Visual Arts	2	20
	Masters in Human Resource Management	1	10
Current residence	On-campus residence	8	80
	Private accommodation	1	10
	Home	1	10
Age of onset of hearing loss	Birth	4	40
	1–3 years	1	10
	4–6 years	4	40
	7 years	1	10
Origin of HL	Acquired	5	50
	Congenital	5	50
Degree of HL	Mild	1	10
	Moderate–severe	1	10
	Severe	2	20
	Not sure	6	60
Types of amplification	Hearing aid	8	80
	Cochlear implant	2	20
Primary communication mode	Total communication	10	100
Parental status	Both hearing	9	90
	Both Deaf	1	10
Communication mode used by parents	Oral	3	30
	Sign language	2	20
	Total communication	5	50
High school educational experience	Inclusive mainstream school	2	20
	Residential school for the Deaf	8	80
School approach/communication option	Sign language	9	90
	Oral approach	1	10

HL, hearing loss.

### Sampling technique

In this research study, purposive sampling was used, as it is well suited to qualitative research designs (Obilor, 2023). This method allows the researcher to select participants’ best positioned to provide insights into the study (Etikan et al., 2016; Thomas, 2022), based on their specific qualities, expertise or familiarity with the phenomenon under investigation (Obilor, 2023).

### Data collection instrument

Semi-structured interviews were conducted, with the use of an interview schedule to gather data. Biographical and case history information was collected, followed by interviews to obtain insights aligned with the study’s objectives. The questions, adapted from Liu (2013), Yang (2021) and Bell and Swart (2018), were divided into four sections: Section A (19 questions) focused on biographical and case history, Section B (3 questions) on educational and instructional adjustments, Section C (5 questions) on psychosocial and cultural adjustments and Section D (3 questions) on physical and environmental adjustments. Probing questions were used to encourage participant reflection. The interviews included open-ended questions (Tuffour, 2017). Each session lasted approximately 45–60 min.

### Data collection procedure

Recruitment efforts included dissemination through the university’s widely utilised notice system and direct engagement with the Disability Unit, where a recruitment poster was displayed. The Disability Unit was also asked to disseminate information about the study directly to students who were registered with disabilities to ensure inclusive outreach. Participants received an information brochure outlining the study’s purpose and requirements. Those willing to participate signed informed consent forms, including permission for the audio recording. Interviews were scheduled at participants’ convenience and conducted in quiet campus locations. Interpreters from the campus networks were engaged and orientated for participants using the Sign language before the study. Participants were encouraged to communicate any special requests in advance to ensure appropriate accommodations were made. Refreshments were provided, and participants received meal vouchers valued at R50 as an incentive. Additionally, a separate R50 incentive was provided to the sign language interpreters who assisted d/DHH participants.

A pilot study was conducted with one HoH participant to assess feasibility, interview suitability and potential challenges. As the participant matched the intended sample characteristics, the feedback confirmed the interview schedule’s effectiveness, and no adjustments were needed. To prevent bias, the pilot study participant was excluded from the main study (Stovner et al., 2014).

### Data analysis

Thematic analysis (Braun & Clarke, 2006) was used to examine the data systematically. Researchers familiarised themselves with the data, generating codes and categories, which led to theme development. The data were transcribed and analysed using NVivo. This qualitative data analysis software facilitated systematic coding and the identification of key themes (Dhakal, 2022). A total of 600 referents resulted in 62 codes, organised into 29 categories and ultimately 11 themes. NVivo enhanced reliability by ensuring consistent coding and enabling pattern recognition.

### Rigour and trustworthiness

The study adhered to credibility, transferability, confirmability and dependability criteria (Ravitch & Carl, 2019). Audio recordings facilitated accurate transcription through comparison with the transcripts, while member checking further enhanced the credibility of the findings (Padgett, 2016). This study prioritises audio recording to uphold participant privacy and comfort. Additionally, ethical approval was granted specifically for audio recordings, reflecting participant preferences for a less intrusive data collection method.

Transferability was considered by documenting participant demographics, behaviours and analysis methods (Ravitch & Carl, 2019). Standardised interview techniques ensured consistency, while negative case analysis and extended interaction with the data enhanced the depth and reliability of the findings. Using fully briefed sign language interpreters further enhanced reliability (Padgett, 2016).

Confirmability was established through an audit trail detailing data analysis stages, ensuring impartiality and representation of participants’ responses (Padgett, 2016). Potential biases were acknowledged and minimised by thoroughly documenting participant experiences and comparing diverse perspectives (Ravitch & Carl, 2019). Dependability was ensured through detailed documentation of data collection and analysis procedures, allowing for study replication. An external audit was conducted to assess the study’s trustworthiness (Padgett, 2016). Although formal confidentiality agreements were not signed by the sign language interpreters in this study, these interpreters were sourced from participants’ personal networks and are bound by professional ethical standards that include strict confidentiality obligations. Prior to the study, interpreters were also briefed on the importance of maintaining participant privacy.

As primary researchers, being final year audiology students, we recognise the importance of being reflective when conducting qualitative research, especially with marginalised groups such as Deaf or HoH students. While we are not d/DHH ourselves, our background in inclusive education and disability studies helped us approach the topic with sensitivity and awareness. To reduce potential bias, we kept a reflexive journal to note our thoughts, assumptions and decisions during the research process. We then went through a peer debriefing process and also discussed our interpretations with our academic supervisor. In addition, member checking was used to ensure that the themes accurately reflected the participants’ views rather than our own, as stated above.

### Data management

The research supervisor was granted access to the consent forms, interview questions and data were securely stored in the supervisor’s office. After 5 years, the data will be shredded and deleted; electronic data will be erased and the hard drive will be formatted. Research data management, which involves the day-to-day administration of data throughout a project, is crucial for enhancing data security and preventing financial, legal and reputational risks for the sponsoring educational institution (Surkis & Read, 2015).

### Ethical and legal considerations

Ethical clearance for the study was obtained from the Humanities and Social Sciences Research Ethics Committee (reference number: HSSREC/00006751/2024), followed by gatekeeper permission from the Registrar of the UKZN. This study was guided by the 2024 version of the World Medical Association’s *Declaration of Helsinki* (WMA, 2025), and all ethical practices were aligned with its recommendations. Ethical considerations were organised according to the four core ethical principles: autonomy, non-maleficence, beneficence and justice. Ethical clearance was obtained prior to the commencement of the study. A comprehensive research protocol was developed and submitted to a reputable ethics committee. Approval was granted after feedback was addressed, and the researchers adhered to the approved protocol throughout the study. Any issues encountered were promptly reported to the committee.

Autonomy was upheld by ensuring that participation was entirely voluntary. All participants were informed of their right to withdraw from the study at any stage without any penalty. Informed consent was obtained, and participants were kept informed of any relevant developments during the research process to ensure transparency. They were also provided with information on support services and were referred to the UKZN Audiology Clinic, if any audiological needs arose.

In line with non-maleficence, data privacy and confidentiality were rigorously protected. Participants were anonymised and assigned numerical labels to protect their identities, and all data were securely stored with access restricted to authorised research team members. The research design and procedures were structured to minimise harm or discomfort to participants. Sensitive topics were approached respectfully, and the researchers ensured that no physical or psychological risks were posed to participants during the study.

Beneficence was demonstrated by prioritising the well-being of participants throughout the research process, with resources for ongoing psychosocial or academic support, where necessary. The study aimed to contribute positively to understanding and improving the experiences of Deaf and HoH students in higher education, particularly in terms of accessibility, support and inclusion.

Finally, justice was promoted by ensuring fair and equal access to participation. The selection of participants was fair and inclusive, without discrimination. Special requirements, such as the need for a South African Sign Language Interpreter (SASL-I), were accommodated through prior arrangements and relevant training. The financial implications of interpreter services were negotiated, and this provision was clearly stated in the recruitment poster to ensure equal opportunity for all potential participants. The researchers had completed online ethics courses, prior to conducting the study. These courses enhanced their understanding of key ethical principles such as informed consent, participant confidentiality and responsible data handling.

## Results and discussion

The results and discussion are organised around the three objectives of the study. A total of 11 themes emerged from the data. Verbatim quotes from participants are presented with the participant number and text extract (TE#).

For Objective 1, which focused on educational or instructional factors, five themes emerged. Objective 2 related to psychosocial and cultural factors, yielded three themes. Lastly, Objective 3, concerning environmental factors, resulted in three themes. These themes are summarised in Table 2.

**TABLE 2:** Themes that emerged in relation to the study objectives.

Objective 1: Educational/instructional factors	Objective 2: Psychosocial and cultural factors	Objective 3: Environmental/physical factors
University support Communication barriers Curriculum rigidity Lecturer attitudes and adaptability Group interactions and social integration	6. Disclosure, counselling and access 7. Transition to university and career choice 8. Mental health, stigma and coping	9. Noise and acoustics 10. Lighting in classrooms 11. Seating arrangements

### Objective 1: Educational/instructional factors

In relation to the first objective, which focuses on instructional and educational factors, five key themes emerged and are discussed below.

#### Theme 1: University support

Overall, participants expressed general appreciation for the support provided by the university and its Disability Unit. Many noted that exam accommodations, interpreters and note-takers were crucial in helping them manage their academic workload. However, the extent and quality of support varied considerably, with some students reporting positive experiences and others highlighting notable limitations.

A key area of support identified was the provision of sign language interpreters, particularly for academic activities. Participants acknowledged the importance of interpreters for accessing lecture content, presentations and tutorials. However, several expressed concern about the insufficient number of interpreters in relation to the students’ needs and the diversity of courses. One participant stated:

‘I have a note-taker as well as an interpreter when I attend lectures. Also when we are in class, we use recording, and after that, I send the recording to my note-taker.’ (P2, Male, 19)

This shortage often resulted in interpreters being available only for lectures, with students left unsupported in other contexts such as residence meetings, group work or extracurricular events. The absence of interpreters’ support in these non-academic spaces contributed to feelings of exclusion and isolation (Andrews et al., 2011). Research also emphasises the importance of support beyond the classroom, including social and residential environments, to foster inclusion (Storbeck et al, 2010). In addition to the shortage of interpreters, several participants noted issues related to the training and preparedness of interpreters, especially in handling the academic content of specific disciplines. University-trained interpreters must be familiar with the subject-specific terminology to provide accurate and effective interpretation in lectures and tutorials (Sarkar & Ghosh, 2024). When interpreters lack this specialised vocabulary, students may miss important information or misinterpret complex academic concepts.

Furthermore, consistent interpreter availability remains a concern. Hameed (2020) recommends providing interpreter support not only during lectures but also after class hours for clarification or academic discussions, which participants indicated could be helpful in enhancing comprehension. Ensuring proficiency in both English and local languages, such as isiZulu, alongside ongoing professional development, is crucial in addressing the diverse linguistic and academic demands of university settings (McGrotty, 2016). These considerations highlight the need for institutions to invest in interpreter training and deployment, particularly in the contexts involving multilingual instruction and technical subject matter.

In some cases, students developed workarounds, such as recording lectures or relying on lip reading, but these methods were not always effective. Additionally, communication breakdowns were noted when interpreters lacked the specialised vocabulary or skills needed for university-level content (Batista & García, 2023), further hindering comprehension and participation.

Another key academic support identified was note-taking services. While some participants appreciated having access to note-takers, others still found it difficult to focus on the interpreter while taking notes, making this support essential (Batista & García, 2023). As one participant (P7) stated, having a note-taker provided valuable academic assistance. Supplementing interpreters with speech recognition software or real-time captioning was suggested as a way to further support students, especially when interpreters were unavailable (Alsalamah, 2020; Hameed, 2020). These adaptive technologies can improve comprehension and accessibility, particularly for technical or fast-paced lectures.

Despite these services, participants also voiced concerns about broader limitations in institutional support. These included lack of awareness of available services, inconsistent communication from the Disability Unit and limited guidance for postgraduate students, as noted by Participant 3. This reflects the inconsistent dissemination of information, which can hinder access to necessary accommodations (Kabtyimer, 2020).

Some students also relied on informal support systems, such as assistance from peers and lecturers in the absence of structured services. While these informal interactions helped bridge some gaps, they often lacked consistency and placed an additional burden on students. This underscores the need for tailored, proactive support services that specifically address the needs of d/DHH students.

Framing this theme within Bronfenbrenner’s Socio-ecological Model, it becomes clear that university support must extend across multiple systems, academic, social and institutional, to effectively promote student development. As Sarkar and Ghosh (2024) argue that higher education institutions can enhance accessibility through a range of accommodations such as real-time transcription, sign language interpretation and accessible learning materials, aligned with participants’ calls for improved awareness, responsiveness and inclusion.

#### Theme 2: Communication barriers

Communication emerged as a central challenge for d/DHH students navigating the university environment. While a few participants shared positive experiences with lecturers who made efforts to accommodate their needs, most described significant barriers to understanding spoken content, particularly during lectures. A common frustration was the fast pace of delivery, which made it difficult to lip-read or follow along, especially in the absence of interpreters. Participant 5 highlighted this challenge:

‘Then the lecturer would, like, speak fast, and I couldn’t actually grasp what he was saying when I was trying to lip-read.’ (P5, Male, 22)

Lip-reading was a common strategy for many students; however, it was often hindered by poor visibility of the speaker’s face, inconsistent lighting or lack of lecturer awareness about facing the student while speaking. These environmental and delivery-related issues frequently impeded access to content and limited student engagement.

To overcome these barriers, participants advocated for simple, practical adjustments by lecturers, including slower speech, clearer articulation, facing the student and regularly checking for understanding. Frumos and Roşu (2019) support these strategies, particularly in settings where interpreters are unavailable. These small but intentional behaviours can significantly enhance accessibility and reduce the communication gap.

Some participants also recommended using technology-based solutions, such as real-time live captioning software, to support comprehension, particularly for technical terms or complex discussions. This aligns with Sarkar and Ghosh’s (2024) call for institutions to adopt real-time transcription tools as part of a broader strategy to improve communication access for students with hearing impairments. Communication difficulties were further compounded in multilingual lectures. One participant described how code-switching between English and isiZulu during instruction created barriers not only for the student but also for their interpreter:

‘The interpreter struggles when the lecturer switches to isiZulu … My interpreter is not isiZulu-speaking.’ (P10, Female, 22)

This highlights the need for institutions to ensure that interpreters are linguistically equipped to navigate diverse lecture environments where more than one language may be used. In such settings, untrained or mismatched interpreters can unintentionally hinder understanding, even when interpreter support is technically present.

While technological aids offer substantial benefits, the presence of well-trained interpreters remain critical. Several participants expressed concern about the varying quality and training levels of interpreters, noting that insufficient skill in academic content or course-specific terminology could lead to miscommunication and hinder understanding. Cheng and Zhang (2017) emphasise that interpreters play a crucial role in bridging communication between d/Deaf students and the hearing academic environment. Without adequate interpreter support, students risk missing important information and becoming disconnected.

This lack of communication support may also contribute to social isolation, as meaningful engagement with lecturers and peers becomes difficult without effective communication channels. As Hyde et al. (2016) suggest, without adequate support, interactions between d/DHH individuals and the hearing world remain limited, heightening the risk of exclusion.

Some participants voiced concerns regarding the overemphasis on hearing aid use by the university support professionals, suggesting that audiological services should focus less on ‘fixing’ hearing and more on supporting communication through inclusive means such as sign language use and communication counselling. This reflects a broader call, echoed by both participants and scholars, such as Storbeck et al. (2010), to move beyond medicalised views of deafness and adopt a culturally sensitive understanding that respects Deaf identity and communication preferences. Ultimately, these findings highlight the need for training academic staff in inclusive communication practices, raising awareness about Deaf culture and investing in adaptive communication support, both human and technological. These efforts are essential for ensuring equitable access to information and fostering a truly inclusive learning environment.

#### Theme 3: Curriculum rigidity

A concern among participants was the rigidity of the undergraduate curriculum, particularly regarding the institutional requirement for all students to complete a compulsory isiZulu language module as part of their degree. This requirement is implemented university wide at the undergraduate level, regardless of a student’s program of study, as part of efforts to promote multilingualism and inclusivity in line with national language policy (Naidoo & Gokool, 2020). However, d/DHH students many of whom primarily use South African Sign Language (SASL) and have had little to no prior exposure to isiZulu expressed confusion, frustration and a sense of exclusion when engaging with this module. The language barrier posed significant learning obstacles, as the course often lacked adapted materials or interpreters proficient in both SASL and isiZulu, making it particularly inaccessible for these student. Participant 7 articulated this sentiment, not merely as a linguistic barrier, but as a clash between the curriculum and Deaf cultural identity:

‘In terms of like deaf culture. They don’t have any knowledge about deaf culture. They must improve. Yes, they teach in sign language, but they have no clue of what is happening, what is the deaf culture. They’re very ignorant. For example, there is a module for IsiZulu and then they force us as deaf people to learn IsiZulu that was strange to me because as I grew up, I never grew up actually learning IsiZulu, it’s not the language that I use; I use sign language; my culture is actually for me to use sign I feel about it.’ (P7, Female, 23)

This quote reflects how the curriculum fails to recognise sign language as the primary mode of communication for many Deaf students, and how compulsory spoken language modules may unintentionally marginalise those from Deaf cultural and linguistic backgrounds.

The issue is not simply about a lack of isiZulu exposure but rather about the incompatibility of certain curriculum requirements with the lived linguistic realities of Deaf students. Some participants described feelings of both confusion and exclusion when expected to learn a language that holds no personal relevance or functional use in their communication.

These findings align with concerns raised in previous studies that emphasise the need for curricula to reflect linguistic diversity and to include flexible pathways for students with different language backgrounds (Bell & Swart, 2018; Nuwagaba & Orech, 2019). In the context of Deaf students, this means recognising SASL as a legitimate language of instruction and communication, and embedding Deaf culture within the curriculum.

Some participants advocated for the introduction of the sign language modules for hearing students and staff, which could promote broader linguistic inclusivity, reduce communication barriers and foster a more respectful and integrated campus environment (Lang & Stinson, 2015). This would also acknowledge the value of SASL not only as an accommodation tool but as a central part of Deaf identity.

Despite the challenges with specific modules, a few participants noted that other parts of the curriculum were more accessible, highlighting the variability in their academic experiences. However, the rigidity of language requirements, particularly the mandatory isiZulu module, remains a key structural barrier for Deaf and HoH students.

#### Theme 4: Lecturer attitudes and adaptability

The participants acknowledged that in some courses, the lecturers were accommodating to their hearing difficulties, while others were not. Inclusivity emerged as a key concept, highlighting participants’ feelings of exclusion from classroom activities. Many d/DHH students reported experiences of indifference from lecturers, who often overlooked their contributions.

This lack of inclusivity in lecture rooms often led to feelings of alienation, as participants reported being left out of communication experiences in lecture rooms because of language barriers. One participant said:

‘Sometimes the lecturers, they ignore d/Deaf people. Even when you raise your hand, they will just not look at you.’ (P9, Female, 25)

Participants commonly recommended that lecturers undergo training in d/Deaf awareness to improve their ability to engage with d/Deaf students in meaningful ways. Many d/Deaf students reported that lecturers lacked the skills to engage effectively, leading to feelings of exclusion. Providing training on Deaf awareness could enhance educator awareness and adaptability, ultimately fostering a more inclusive environment that supports academic participation and success. Educators and administrators both played a critical role in shaping the experiences of students with hearing impairments (Sarkar & Ghosh, 2024).

Participant 2 stated that the university should conduct more campaigns with lecturers about understanding deafness, deaf culture and communicating with the d/Deaf. Another participant said:

‘I would actually like maybe, make a deaf awareness and lecturers be aware about deaf student around them, and then be aware in their classes there are deaf people. And then, being social around deaf people, not exclude or isolate deaf people.’ (P6, Female, 23)

This emphasises the need for greater awareness of Deaf identity and culture within the South African educational institutions. Nuwagaba and Orech (2019) found that the quality of interactions and support between lecturers and students who are d/DHH plays a critical role in these students’ academic adjustment in Uganda, highlighting the broader need for institutional support. A significant barrier to inclusion is the lack of understanding among peers and educators about the challenges faced by those with hearing impairments (Ashraf et al., 2023). A comprehensive approach is needed to address this, including raising awareness, promoting inclusive practices and offering necessary accommodations (Tufar, 2024).

#### Theme 5: Group interactions and social integration

The diverse experiences regarding group interactions and social integration highlighted the complex social dynamics that d/DHH students face. Participants who reported positive experiences emphasised the importance of supportive hearing peers who were willing to engage and accommodate their needs:

‘Sometimes there are positive things, when I communicate with them, I don’t feel rejected, they want to learn, we joke, we have fun, they ask questions and they want to learn, they use gestures and later on when you meet them, they tell stories, chat to me and you feel positive. I feel positive about myself, and it boosts my confidence that they’re interested to interact with me.’ (P8, Female, 21)

Efforts by hearing peers to communicate effectively, such as transcribing conversations or learning basic sign language, could foster meaningful cross-cultural interactions and contribute to an inclusive environment that values Deaf culture (Williams & Chang, 2016). This proactive behaviour not only aids communication but also enhances mutual respect. Positive social exchanges are crucial for the psychological well-being of Deaf students (Kishida et al., 2022). Support from Deaf peers, as noted by Participant 10, creates a sense of belonging and solidarity, reinforcing community among Deaf students (Oliva et al., 2016). However, exclusion because of a lack of understanding and awareness among hearing peers remains a significant issue, aligning with findings from Bell and Swart (2018) and Nuwagaba and Orech (2019). These challenges are particularly evident in group work, where d/Deaf students struggle with collaboration, especially in online settings, as noted by Participant 8:

‘I felt rejected and as if they were just looking down on me and it wasn’t a very good experience.’ (P8, Female, 21)

‘Yeah. I kind of feel frustrated. Because at most time, I am fighting to have access to information. Which is not there. Not much. Because, for example, most modules that I do have WhatsApp groups where they are students. And even though they are aware that there’s a deaf student who cannot access audio, there’s still that’s voice notes thing that still continues to happen instead of typing.’ (P1, Female, 23)

Echoing participants’ frustrations with group work, Frumos and Roşu (2019) similarly recommend strategies to enhance group dynamics for d/DHH students, including limiting group sizes, working in smaller groups for clearer communication, sitting in a circle for improved visual contact and ensuring one speaker at a time. Slowing the conversation pace and allowing extra time for information processing are also crucial for fostering inclusivity. Furthermore, listening to d/Deaf students’ preferences and involving d/Deaf university teachers as mentors can help create individualised and more effective communication strategies. This support and collaboration are consistent with the Bronfenbrenner Model.

### Objective 2: Psychosocial and cultural factors

In addressing the second study objective, examining psychosocial and cultural influences on the adjustment of Deaf and HoH students at the UKZN, three central themes were identified.

#### Theme 6: Disclosure, counselling and access

Eight participants disclosed their hearing impairment during the university application process, leading to the establishment of support services, including interpreters and note-takers. These participants received assistance through the Disability Unit, despite a shortage of interpreters. Early disclosure was essential for obtaining timely accommodations, positively impacting academic success (Lin & Miloň, 2022) as discussed earlier in **Theme 1**. However, some participants reported insufficient support, feeling neglected and having to navigate challenges on their own. A lack of awareness and support could contribute to feelings of marginalisation (Lang & Stinson, 2015). Peer support also played a significant role, as some participants were guided to the Disability Unit by others who used the sign language. Non-disclosure or late disclosure often resulted in isolation, especially for those transitioning from deaf schools to mainstream settings (McDaid, 2013). The study found varied responses to counselling services, with six participants opting not to attend because of stigma, perceived ineffectiveness or lack of accommodations (McCay & Goodman, 2015). Those who did attend reported positive experiences, particularly when services were accessible (Scherer, 2016), while others found the sessions lacking in meaningful dialogue (Rotheram-Borus et al., 2014).

The study also explored the complexities of self-identity for d/Deaf students, particularly their duality of identifying with the Deaf community while seeking acceptance in the hearing world (Bell et al., 2016). Students who disclosed their impairment voluntarily benefited from appropriate academic support, while those who avoided disclosure faced educational challenges (Sekoto & Hlayisi, 2023). Many students disclosed their disability on application forms because of legal requirements but preferred not to highlight it unless necessary to avoid stigma (Bell & Swart, 2018).

#### Theme 7: Transition to the university and career choices

Regarding educational background, most participants attended deaf schools, where inclusive environments facilitated a sense of community and eased their transition to the university. However, one participant from a mainstream school found the transition more difficult, highlighting the need for improved support for students from diverse educational backgrounds (Lang & Stinson, 2015). The study emphasised the importance of a nurturing environment for academic and emotional success, reducing feelings of isolation and strengthening peer relationships. Personal experiences with deafness influenced career choices. Some participants chose careers to give back to the Deaf community, while others opted for less communication-intensive roles because of challenges in interpersonal communication (Storbeck et al., 2010). Several participants selected courses based on passion rather than their hearing impairment, demonstrating that d/Deaf students can excel in diverse fields when provided with proper support (Kishida et al., 2022).

#### Theme 8: Mental health, stigma and coping

This theme explores the emotional and psychological impact that communication barriers, social isolation and lack of accommodations (e.g. sign language interpreters and captioning) have on the university students who are d/Deaf. Participants attributed feelings of frustration, loneliness and low self-esteem to these challenges, emphasising how such barriers negatively affect their mental health. Many students experience frustration, loneliness and depression because of limited social interactions, which affects their self-esteem and well-being.

However, some students demonstrated resilience, emphasising that external environmental barriers rather than Deafness were the primary challenge. Coping strategies varied, including journaling, mindfulness techniques and participation in extracurricular activities such as sports, which helped reduce stress and foster social connections.

Participants reported that misconceptions, biases and stigma around deafness and Deaf culture persisted in both academic and social settings. These biases, often stemming from peers, educators and even family members, include the harmful belief that d/Deaf individuals have limited cognitive abilities and are incapable of performing certain academic tasks such as reading and writing. Such misconceptions can result in missed academic and social opportunities for students.

Family attitudes also played a crucial role in shaping self-confidence and identity, with supportive families fostering resilience, while unsupportive families contributed to feelings of alienation. One participant, reflecting on their own experience of limited familial support, shared:

‘Sometimes when your family is hearing, they will think like you you’re not part of the family. They will think you are monkey, and the language itself. Okay. The family sometimes (P6, Female, 23)

The alienation experienced by d/Deaf individuals is often because of families not fully understanding or accepting Deaf culture and language. Supportive families foster a strong sense of identity and belonging, while those with misconceptions can lead to isolation and low self-esteem (Ashraf et al., 2023). Comparing communication using the sign language to ‘monkey language’ reflects disrespect for Deaf culture, causing emotional distress. According to Bronfenbrenner’s Model, families are an important part of the microsystem, and early family interactions are crucial in shaping self-worth, community belonging and socialisation (Williams & Chang, 2016).

Engagement in university extra-curricular activities at the university contributed meaningfully to students’ stress management and overall well-being. Two participants were excited to engage in the university sporting activities (netball and soccer) as it relieved some of the stresses they were experiencing. However, this initially surprised the hearing individuals:

‘Yes. Most definitely. I am part of the netball team. And, you know, the hearing students were very shocked when I was part of that team because they were like, how? You can, you are able to actually do things even though you are deaf. So yeah … Because my disability doesn’t mean that I cannot do things. I’m actually capable. So, it was good to actually raise some sort of awareness for them in terms of that.’ (P1, Female, 23)

Participation in the university activities like sports and advocacy campaigns was a positive experience for some students, helping break stereotypes and raise awareness about d/Deaf students’ capabilities. Others preferred solitude or had not yet engaged in activities because of personal preference or being new to university life.

The findings emphasise the need for the universities to foster inclusive environment through cultural competence training, improved accessibility measures and awareness programmes that challenge misconceptions about deafness (Williams & Chang, 2016).

### Objective 3: Environmental/physical factors

Three key themes emerged in response to the third study objective of exploring the *environmental/physical factors* influencing the adjustment of students who are Deaf or HoH attending the UKZN.

#### Theme 9: Noise and acoustics

Many participants reported that background noise, particularly from other students talking, made it difficult to focus on interpreters and follow lectures. Distractions from the classroom environment further disrupted communication and comprehension (Jamieson et al., 2018). One participant (P7) described the challenge of distractions during lectures, noting how noise from the back of the room interfered with their ability to follow the lecture content. One participant noted that their seating arrangement helped mitigate some of the noise issues, but overall, students expressed frustration with the lack of noise control. Research supports these concerns, emphasising that excessive noise interferes with interpretation and reduces access to information. Suggestions to improve the learning environment include soundproofing classrooms or creating quiet zones for group activities (Guardino & Antia, 2012). To address these challenges, the universities should assess and work towards reducing excessive classroom noise levels. Rather than costly soundproofing, more feasible sound treatment strategies such as installing acoustic panels, using carpets or curtains to absorb sound and minimising background noise could be considered, especially in smaller lecture venues. In large lecture halls, priority could be given to individualised support for d/DHH students such as preferential seating, captioned recordings or assistive listening devices, which are more practical and cost-effective in the South African higher education context (Ohba & Malenya, 2022):

‘Sometimes it’s bad in the classroom when you try to focus on the interpreter, but the students in the back are talking … It affects our communication; the lecture is saying something, but there is noise, and sometimes there is a lot of distraction.’ (P7, Female, 23)

#### Theme 10: Lighting in classrooms

Several participants expressed concerns about classroom lighting. One participant discussed the challenges of classrooms with dimmed lights when lecturers used projectors, which interfered with the visual nature of sign language:

‘Some lecturers use a projector and turn off the lights, and since it’s a visual language, it becomes a challenge.’ (P1, Female, 23)

#### Theme 11: Seating arrangements

Seating arrangements were found to significantly influence the ability of d/DHH students to access communication and engage with course content. Most participants reported that sitting in the front of the classroom, close to the interpreter, was essential for clear communication. For instance, one participant stated that being in the front was essential for acquiring all necessary information:

‘As a d/Deaf student, I need the interpreter to be in front of me. I cannot sit at the back because I won’t have any access to any communication.’ (P8, Female, 21)

This highlights the critical role of seating arrangements in ensuring that d/DHH students have a clear line of sight to the interpreter, which is essential for following the lecture content and participating actively in class discussions.

This is in line with recommendations by Hameed and Ul-Ain (2020), who suggest reserving the first rows for students with special communication needs, such as d/DHH, to improve interaction and ensure clear sightlines to interpreters. Strategic seating arrangements are crucial for optimising visual access to interpreters (Alasim, 2018), and the universities should prioritise such accommodations to enhance learning experiences (Braun et al., 2018).

Consistently implementing and strictly enforcing preferential seating policies for d/DHH students is crucial to ensuring that their communication needs are met and that they are not disadvantaged by others occupying designated seats (Bell & Swart, 2018). However, the study revealed that these policies were often enforced inconsistently, with some participants reporting that other students frequently occupied their assigned seats, thereby negating the intended benefit of the accommodation.

## Limitations

The analysis of qualitative data is inherently shaped by researchers’ backgrounds, perspectives and experiences, which may influence interpretation. To mitigate potential bias, the research team engaged in collaborative discussions to refine emerging themes and supported findings with direct participant quotations, thereby enhancing credibility and reflecting the richness of participants’ experiences. The use of self-reported data presents inherent limitations, including the potential for bias arising from social desirability, memory recall and subjective interpretation, all of which may affect the accuracy and completeness of the data collected. The inclusion of sign language interpreters introduced challenges such as possible loss of meaning or interpreter influence, particularly where interpreters were affiliated with participants. To address this, interpreters received pre-interview training focused on maintaining neutrality and adhering to the study’s objectives. Although the use of an independent interpreter was preferred, financial constraints made this unfeasible. As the study was conducted solely at the University of KwaZulu-Natal, the findings may have limited generalisability to other institutional contexts. Furthermore, the necessary reliance on interpreters may have impacted the depth and authenticity of participant expression, potentially influencing the overall quality of the data.

## Conclusion

This study aimed to explore the factors affecting the adjustment of d/DHH students at the University of KwaZulu-Natal, focusing on instructional, psychosocial and environmental influences. The findings revealed several significant challenges. These included variable levels of support – where accommodations were occasionally available, but inconsistently applied – limited access to qualified interpreters and inadequate note-taking assistance. Participants also reported negative attitudinal barriers, particularly from peers and lecturers, which adversely affected their academic and social integration.

Environmental factors, such as high classroom noise levels, inadequate lighting and unreliable preferential seating arrangements, further impeded effective communication and learning. Recommendations put forth by the study findings include the implementation of targeted strategies, accommodations and assistive technologies to enhance the academic and social experiences of the d/DHH students. These may include the use of written summaries, visual aids and interactive teaching methods to support diverse learning needs. The consistent provision of qualified sign language interpreters and lecture-related accommodations by lecturers is also essential in fostering a more inclusive educational environment.

By adopting such measures, the universities can help promote equitable opportunities for success while demonstrating a strong commitment to diversity, accessibility and inclusive higher education.

## Footnotes

1 The World Health Organization (WHO) describes ‘deaf’ as a profound hearing impairment characterised by a complete loss of hearing in one or both ears, with a hearing threshold of 81 dB or higher (WHO, 2024). The term ‘HoH’ refers to a condition in which individuals experience varying degrees of hearing loss, making it difficult to perceive sounds, speech or specific frequencies (WHO, 2024). The term ‘Deaf’ (capitalised) typically refers to individuals who identify with the Deaf community and culture, while ‘deaf’ (lowercase) refers to the audiological condition of hearing loss (Cooper, 2019). Individuals classified as Deaf or HoH often face challenges in understanding conversations, particularly in environments with high ambient noise levels (WHO, 2024).
